# Long-term tumor remission under trastuzumab treatment for HER2 positive metastatic breast cancer – results from the HER-OS patient registry

**DOI:** 10.1186/1471-2407-14-806

**Published:** 2014-11-04

**Authors:** Isabell Witzel, Volkmar Müller, Wolfgang Abenhardt, Manfred Kaufmann, Winfried Schoenegg, Andreas Schneeweis, Fritz Jänicke

**Affiliations:** Department of Gynecology, University Medical Center Hamburg-Eppendorf, Martinistrasse 52, D- 20246 Hamburg, Germany; Onkologische Praxis Elisenhof, Munich, Germany; Department of Obstetrics and Gynecology, University Hospital, Frankfurt, Germany; Breast Center, DRK Hospital, Berlin, Germany; Department of Obstetrics and Gynecology, University Hospital, Heidelberg, Germany

**Keywords:** HER2, Metastatic breast cancer, Trastuzumab

## Abstract

**Background:**

In this study, we examined patients who had non-progressive disease for at least 2 years after diagnosis of inoperable locoregional recurrent or metastatic breast cancer under continuous trastuzumab treatment. Our primary goal was to assess the long-term outcome of patients with durable response to trastuzumab.

**Methods:**

268 patients with HER2-positive inoperable locally recurrent or metastatic breast cancer and non-progressive disease for at least 2 years under trastuzumab treatment were documented retrospectively or prospectively in the HER-OS registry, an online documentation tool, between December 2006 and September 2010 by 71 German oncology centers. The study end point was time to tumor progression.

**Results:**

Overall, 47.1% of patients (95% confidence interval (CI): 39.9–54.1%) remained in remission for more than 5 years, while the median time to progression was 4.5 years (95% CI: 4.0–6.6 years). Lower age (<50 years) and good performance status (ECOG 0) at time of trastuzumab treatment initiation as well as complete remission after initial trastuzumab treatment were associated with longer time to progression. Interruption of trastuzumab therapy correlated with shorter time to progression.

**Conclusions:**

HER2-positive patients, who initially respond to palliative treatment with trastuzumab, can achieve a long-term tumor remission of several years.

## Background

Although the majority of breast cancer patients can be cured of their disease, up to 20% will develop metastatic breast cancer (MBC). The Human Epidermal Growth Factor Receptor 2 (HER2) is overexpressed or amplified in 15% of breast tumors [1] and is associated with a more aggressive tumor behavior, shorter disease-free and overall survival [2–4]. Trastuzumab (Herceptin®), a monoclonal antibody directed against HER2, has shown to improve survival in combination with chemotherapy compared to non-trastuzumab-based treatment [5–7]. It has therefore become the standard treatment in adjuvant and metastatic HER2-positive breast cancer. Although the majority of patients with MBC treated with trastuzumab-based regimens progress within one year [5, 8], few patients experience prolonged remission [9, 10]. Limited data have been published on long term remission under treatment with trastuzumab and are usually based on case reports or small patient numbers [11–13]. Aspects such as clinical predictive factors of long-term response to trastuzumab or the optimal duration of trastuzumab therapy in MBC patients achieving stable response remain to be reported. The primary goal of this study was to assess the long-term outcome of patients with durable response to trastuzumab. In addition, factors that could be associated with long-term tumor remission under trastuzumab were identified in an exploratory analysis.

## Methods

### Selection of patients

Patients with HER2 positive inoperable locally recurrent or metastatic breast cancer and non-progressive disease for at least 2 years under continuous trastuzumab therapy (complete or partial response or stable disease) met the inclusion criteria to be documented in the HER-OS database. Positive HER2 status was defined as immunohistochemistry (IHC) staining of 3+ or immunohistochemistry staining of 2+ and positive fluorescence in-situ hybridization (FISH, HER2/CEP17 ratio >2.2). Between December 2006 and September 2010, 447 patients under trastuzumab treatment were documented in 71 German medical centers within the HER-OS database, an online- documentation platform for patients with advanced HER2 positive breast cancer. The database for the register was set up by an review board (see Authors’ contributions) as a collection of case reports. The project fulfilled the criteria of a non-interventional study according to the European Community and German legislation, and therefore required no ethical committee vote [14]. Patients gave informed consent to have their medical records reviewed according to the review board guidelines. The not publicly available HER-OS database (owner: Roche Pharma AG, Germany) included documentation of demographic data, clinico-pathological data of the primary tumor, treatment strategies and concomitant diseases. Treatment with trastuzumab, further antineoplastic therapies and tumor status were documented every 6 months after treatment initiation with trastuzumab. Retrospective as well as partial retro-/prospective documentation was allowed. Patient data was anonymized.

The study end point was time to tumor progression (TTP). The HER-OS database was closed in September 2010. The observation period until disease progression or end of study was 41.2 months (median; range: 24.3–117.1 months).

Only 268 of 447 patients (60.0%) had complete documentation of prior treatments, met the inclusion criteria, were without progression for at least 2 years after the initiation of trastuzumab treatment, and were therefore considered eligible for further analyses.

### Treatment

Since this study was non-interventional, patients were treated at their physician’s choice. The physicians chose trastuzumab treatment intervals and dosages as well as combination of trastuzumab with other chemotherapeutic or endocrine treatments. The physicians also determined cardiac monitoring intervals, which were mostly performed in 6-monthly intervals.

### Statistical methods

Statistical analysis focused on the summary and detailed description of the data. Unless otherwise stated, percentages were displayed as adjusted values, so that patients with missing data were not taken into account.

Primary outcome variable of the study was time to tumor progression (TTP). Nonparametric estimates of survivor functions were calculated by the Kaplan-Meier method. Differences in time distributions were analyzed using Peto’s logrank test. In a multivariate analysis including all parameters of the univariate analysis, forward selection was performed with an entry of 0.25 and a stay of 0.15. Results were regarded as statistically significant at a p-value ≤0.10. Analyses in subgroups were made post-hoc and should be seen as exploratory. The statistical analysis was performed with the program SAS™ version 9.2.

## Results

### Patients’ characteristics

The median age at diagnosis of breast cancer was 53.8 years (range 29–86 years). 27.2% of patients (n = 64) had metastases at first diagnosis of breast cancer. 37.5% of patients (n = 94) were hormone receptor negative. Of those patients with non-metastatic disease at diagnosis, 76.6% (n = 131) had received chemotherapy in the adjuvant setting, 42.7% (n = 73) a taxane containing regimen. 50.9% (n = 87) had received adjuvant endocrine treatment, 8.2% (n = 14) adjuvant trastuzumab treatment. The median disease-free survival for 131 patients with non-metastatic disease at diagnosis amounted to 3.3 years (range: 1 month – 15.5 years).

At the time of disease recurrence or onset of metastatic disease, 15.3% (n = 41) of the 268 women suffered from inoperable locoregional recurrent disease, 52.2% (n = 140) from distant metastases and 32.5% (n = 87) from both. Metastases were predominantly found in bone (n = 102; 38.1%), liver (n = 88; 32.8%), and lung (n = 75; 28.0%). Brain metastases were present in only 2.2% of documented patients (n = 6).

In general, patients had a good performance status at the beginning of trastuzumab therapy. 49.8% (n = 115) had Eastern Cooperative Oncology Group (ECOG) performance status 0. 42.2% (n = 108) of patients had normal weight, while 51.6% (n = 132) were overweight or obese, and 6.3% (n = 16) were underweight.

Nearly every fifth patient (19.4%) presented with relevant comorbidities, which were mostly hypertension (10.1% of all patients) or cardiac arrhythmia (2.6% of all patients).

Tables 1 and 2 provide detailed patients’ characteristics at the time of diagnosis of breast cancer and at the start of palliative trastuzumab treatment.

**Table 1:** **Patient characteristics at diagnosis of breast cancer**

Characteristics	Number	%
Age, years		
Median (Range)	*53.8 (29–86)*	
TNM status		
T0-T1	*77*	*30.4*
T2	*119*	*47.1*
T3 and T4	*57*	*22.5*
N0	*74*	*31.0*
N1-N3	*165*	*69.0*
M0	*171*	*72.8*
M1	*64*	*27.2*
Grading		
G1	*11*	*4.4*
G2	*121*	*48.4*
G3	*118*	*47.2*
HER2 status		
+2 and positive FISH	*11*	*4.1*
+3	*257*	*95.9*
Estrogen receptor (ER)/progesterone receptor (PR)		
ER positive/PR positive	*105*	*41.8*
ER positive/PR negative	*38*	*15.1*
ER negative/PR positive	*14*	*5.6*
ER negative/PR negative	*94*	*37.5*
ER or PR unknown	*17*

**Table 2:** **Disease status before initiation of trastuzumab treatment**

Characteristics	Number	%
Age (years)		
Median (Range)	*58.5 (31–86)*	
BMI (kg/m^2^)		
Median (Range)	*25.1 (16–50)*	*6.3*
<18	*16*	*42.2*
18–25	*108*	*51.5*
>25	*132*	
ECOG performance status		
0	*115*	*49.8*
1	*108*	*46.8*
2	*8*	*3.4*
Disease-free survival (months)		
Median (Range)	*37.3 (1–413)*	
Site of disease recurrence		
Inoperable locoregional recurrence	*41*	*15.3*
Metastatic disease	*140*	*52.2*
Both	*87*	*32.5*
Site of locoregional recurrence		
Breast	*47*	*17.5*
Axillary lymph nodes	*64*	*23.9*
Supraclavicular lymph nodes	*33*	*12.3*
Chest wall	*23*	*8.6*
not specified	*15*	*5.6*
Site of metastatic disease		
Lung	*75*	*28.0*
Liver	*88*	*32.8*
Bone	*102*	*38.1*
CNS	*6*	*2.2*
Other	*25*	*9.3*

### Trastuzumab treatment

Although the administration of trastuzumab as weekly and three-weekly infusion was balanced at the beginning of palliative treatment, the majority of patients switched to three-weekly intervals during therapy (84.0%).

In 78.4% of women, trastuzumab was started in a combination treatment with chemotherapy (n = 169; 63.1%) or with endocrine treatment (n = 92; 34.3%). The most commonly used chemotherapeutic agents were taxanes (40.3% total; 20.9% paclitaxel; 19.4% docetaxel), vinorelbine (22.4%) and antimetabolites (17.2% total; 12.7% capecitabine; 2.6% gemcitabine; 1.9% fluorouracil). Anthracyclines were also combined with trastuzumab in 2.6% of women (2.2% doxorubicin; 0.4% epirubicin). In case of endocrine therapy in combination with trastuzumab, patients mostly received an aromatase inhibitor or tamoxifen. In 21.6% of women (n = 58), trastuzumab was used as single agent.

### Interruption of trastuzumab therapy

17 patients (6.4%) received lower trastuzumab dosages due to therapy interruption, mostly due to other illnesses. The most frequent reasons for interruption of trastuzumab therapy were patient’s wish (1.5%) or cardiac adverse event (1.1%). Patients who experienced progression within 6 months after trastuzumab interruption (n = 3) were excluded from further analyses.

### Response to trastuzumab

A clinical response to trastuzumab treatment was required in order to be included in the study. 38.7% of patients (n = 103) had complete remission, 32.0% (n = 85) partial remission, and 29.3% (n = 78) stable disease as best response to trastuzumab treatment. A remission (complete or partial) was documented after a median time of 7.3 months since treatment initiation.

**Figure 1:**
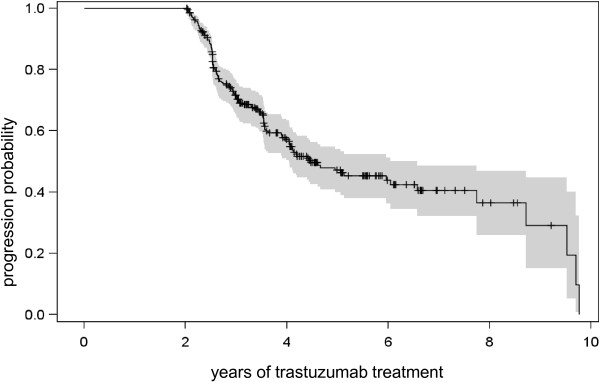
**Probability of progression during palliative trastuzumab treatment.**

Until the end of the study, 126 patients (47.0%) had progressive disease during continuous trastuzumab treatment with an estimated median time to progression (TTP) of 4.5 years (95% CI: 4.0–6.6 years). It was estimated that 47.1% of patients (95% CI: 39.9–54.1%) remained in remission for more than 5 years, 40.5% (95% CI: 32.1–48.7%) for more than 7 years, and 29.2% (95% CI: 15.1–44.8%) for more than 9 years (Table 3, Figure 1).

**Table 3:** **Time to tumor progression at the end of each year during follow-up**

Year	Patients with progression	Patients censored	Product-limit survival estimates	95%-confidence interval of survival estimates		Patients left
				Lower limit	Upper limit	
**1 and 2**	0	0	100.0%			268
**3**	72	29	71.6%	65.6%	76.8%	167
**4**	29	39	57.1%	50.4%	63.3%	99
**5**	15	29	47.1%	39.9%	54.1%	55
**6**	3	22	43.9%	36.2%	51.3%	30
**7**	2	15	40.5%	32.1%	48.7%	13
**8**	1	4	36.5%	26.0%	47.0%	8
**9**	1	3	29.2%	15.1%	44.8%	4
**10**	3	1	0.0%	-	-	0

### Factors associated with long-term tumor remission

Patients who were younger (age <50 years) at diagnosis of inoperable locoregional recurrent or metastatic disease and had a good performance status (ECOG 0) exhibited a trend towards longer TTP in the univariate analysis (p = 0.07 and p = 0.08, respectively; Table 4). In addition, best response to trastuzumab treatment had an influence on TTP (p = 0.057; Table 4). Interruption of trastuzumab treatment was associated with shorter TTP (p = 0.0005; Table 4). We could not observe an influence of tumor size, grading, hormone receptor status, nodal status or disease-free survival in univariate analysis. The absence of distant metastases at the onset of trastuzumab treatment or the initial combination of trastuzumab with endocrine therapy or chemotherapy had also no impact on TTP (Table 4).

**Table 4:** **Association of clinicopathological and treatment characteristics with time to tumor progression (univariate analysis)**

Parameter		Patients (total)	Patients (progressed)	Time to progression (years)		p-value
TNM status at diagnosis	T0-T1	*77*	*28*	5.10	4.13- n.e.	*0.1423*
	T2-T4	*176*	*92*	4.06	3.56-7.74	
	N0	*74*	*37*	4.11	3.30-6.05	*0.2813*
	N1-N3	*165*	*75*	5.06	4.05-9.76	
	M0	*171*	*79*	5.06	4.05-9.70	*0.2367*
	M1	*64*	*31*	4.39	3.51-6.58	
Grading	G1-G2	*132*	*63*	4.13	3.55-6.05	*0.3122*
	G3	*118*	*55*	5.06	4.04-9.53	
Disease-free survival	0-5 years	*203*	*91*	5.12	4.11-8.72	*0.2183*
	>5 years	*56*	*32*	3.46	3.00-4.44	
Hormone receptor status	ER negative	*109*	*48*	5.96	4.04-9.53	*0.1876*
	ER positive	*148*	*73*	4.13	3.55-6.05	
	PR negative	*132*	*61*	5.06	4.05-6.58	*0.6547*
	PR positive	*120*	*56*	4.11	3.53-9.70	
	ER and PR negative	*94*	*42*	5.06	4.04-9.53	*0.3789*
	ER or PR positive	*157*	*75*	4.18	3.56-6.58	
Age at trastuzumab start	< 50 years	*70*	*32*	5.96	4.11-9.76	*0.0744*
	≥50 years	*198*	*94*	4.20	3.56-6.05	
ECOG status at trastuzumab start	0	*115*	*54*	4.61	3.91-9.76	*0.0812*
	1-4	*116*	*59*	4.05	3.44-5.96	
Site of recurrence at trastuzumab start	locoregional					*0.1156*
	recurrent only	55	22	9.70	3.63-9.70	
	Bone metastases only	*41*	*21*	4.05	3.30-n.e.	
	Visceral metastases	139	69	4.39	3.55-6.58	
Initial response to trastuzumab treatment	Complete remission	*48*	*16*	8.72	4.46-9.76	*0.0571*
	Partial remission	*80*	*46*	4.03	3.50-4.41	
	Stable disease	*82*	*37*	4.66	3.56-n.e.	
Interruption of trastuzumab treatment	no therapy interruption	*251*	*109*	5.13	4.11-9.53	*0.0005*
	therapy interruption	*14*	*14*	3.51	2.36-4.44	

In the multivariate analyses, interruption of trastuzumab treatment turned out to be associated with shorter TTP (p = 0.0015; data not shown).

## Discussion

In our group of patients responding at least 2 years to trastuzumab treatment for inoperable locoregional recurrent or metastatic breast cancer, long-term tumor remission for several years could be achieved. The median time to progression in our study cohort was 4.5 years. We could identify that younger age (under the age 50) and good performance status (ECOG 0) at the initiation of trastuzumab treatment were associated with longer TTP. An interruption of trastuzumab treatment correlated with shorter TTP in univariate and multivariate analysis.

Trastuzumab therapy has increased response rates and survival times in the metastatic setting [15]. We can report a complete remission rate of 38.7% in HER2 positive patients receiving trastuzumab treatment for advanced breast cancer. To our knowledge, we are the first to describe a clinical cohort of patients with advanced breast cancer disease who benefit from trastuzumab treatment for several years.

Long-term follow-up, beyond 3 to 5 years, is exceptional in the metastatic breast cancer literature as median survival ranges between 2 and 4 years. While most patients with metastatic breast cancer respond transiently to conventional treatments, the majority develop evidence of progressive disease within 12 to 24 months of first-line therapy [16, 17]. However, some patients who achieve a complete remission after chemotherapy remain in this state for prolonged periods of time, with some even beyond 20 years [18, 19]. Tomiak et al. reported that 20% of metastatic breast cancer patients who achieved complete remission with chemotherapy were alive and without disease progression for more than 5 years [19]. In another retrospective analysis of 147 premenopausal women with metastatic breast cancer receiving chemotherapy, 28% of patients were reported to be alive after a follow-up period of 5 years [20]. In a cohort of 1581 metastatic breast cancer patients treated with anthracycline containing therapy for a maximum of 2 years in the 1970s and 1980s, complete remission rates of 16.6% were described. 3% of patients remained in complete remission for more than 5 years [18]. The long-term survivors described in those studies were usually young, had excellent performance status and limited metastatic disease. In line with these results, we were able to show that of all clinicopathological parameters, age at initiation of trastuzumab treatment (under age 50), good performance status (ECOG 0) and initial response to trastuzumab treatment (complete remission) were associated with longer TTP. However, also patients with only partial or stable disease had long-term tumor remission. Interestingly, in our group of patients, site of disease recurrence was not associated with TTP (inoperable locoregional disease recurrence vs. bone metastases vs. visceral metastases).

As our data base was documented in several institutions, it reflects the reality of medical care of HER2 positive MBC patients between 2006 and 2010. Most of the patients had received trastuzumab in combination with chemotherapy or endocrine therapy while 20% of patients had received trastuzumab only as monotherapy. According to published data, trastuzumab as single-agent first-line treatment in MBC showed efficacy. 57% of responding patients had stable disease longer than 12 months [6]. In HER2 positive MBC patients who progressed under at least one cytotoxic regimen, response rates of 48% (19% complete or partial remission, 29% stable disease) with trastuzumab monotherapy were reported [21]. In this trial, one third of patients lived for more than three years with trastuzumab monotherapy.

We were able to demonstrate that therapy cessation or interruption should be avoided as it was associated with shorter TTP in our patient cohort. Continuous suppression of the HER2 pathway may be important, and was already demonstrated by the benefit of trastuzumab use beyond progression [22]. Our data is supported by a retrospective cohort of 84 patients treated with trastuzumab for MBC in two different institutions. One institution stopped trastuzumab treatment after two years of response, in this institution durable response rates were lower than in the institution that continued trastuzumab treatment after two years of response (durable response rates 6 versus 11%) [10]. Although there might be a benefit of longer trastuzumab treatment in the metastatic setting, the duration of trastuzumab treatment is still unclear.

The study was designed to find new hypotheses regarding long-term remission. Therefore a significance level of p < 0.1 seemed appropriate. A lower p-value of p = 0.05 as used in randomized clinical trials could have led to the exclusion of hypotheses that might be worth to evaluate further. A drawback of our study is that it is a single-arm multicenter study with no comparative cohort, so that we were not able to draw unequivocal conclusions but could only describe parameters influencing long-term remission within a highly selected sample. It is important to note that we report only time to disease progression and that we have not collected data about overall survival. However, overall survival rates can be expected to be significantly longer than the TTP reported here because several anti-HER2 treatment strategies can still be applied in HER2 positive metastatic breast cancer after disease progression.

## Conclusions

Although the fraction of metastatic breast cancer patients with long-term tumor remission is small, we provide evidence that HER2 positive patients who initially respond to palliative treatment with trastuzumab can achieve a long-term tumor remission of several years.
